# The validity of using ICD-9 codes and pharmacy records to identify patients with chronic obstructive pulmonary disease

**DOI:** 10.1186/1472-6963-11-37

**Published:** 2011-02-16

**Authors:** Colin R Cooke, Min J Joo, Stephen M Anderson, Todd A Lee, Edmunds M Udris, Eric Johnson, David H Au

**Affiliations:** 1 Division of Pulmonary and Critical Care Medicine, University of Michigan, Ann Arbor, MI, USA; 2 Robert Wood Johnson Foundation Clinical Scholars Program, University of Michigan, Ann Arbor, MI, USA; 3 Center for Management of Complex Chronic Care, Edward Hines Jr. VA Hospital, Hines, IL, USA; 4 Section of Pulmonary, Critical Care, Sleep and Allergy, University of Illinois at Chicago, Chicago, IL, USA; 5 Health Services Research and Development, VA Puget Sound Health Care System, Seattle, WA, USA; 6 Center for Pharmacoeconomic Research and Department of Pharmacy Practice, College of Pharmacy, University of Illinois at Chicago, Chicago, IL, USA; 7 Group Health Research Institute, Seattle, WA, USA; 8 Division of Pulmonary & Critical Care Medicine, University of Washington, Seattle, WA, USA

## Abstract

**Background:**

Administrative data is often used to identify patients with chronic obstructive pulmonary disease (COPD), yet the validity of this approach is unclear. We sought to develop a predictive model utilizing administrative data to accurately identify patients with COPD.

**Methods:**

Sequential logistic regression models were constructed using 9573 patients with postbronchodilator spirometry at two Veterans Affairs medical centers (2003-2007). COPD was defined as: 1) FEV1/FVC <0.70, and 2) FEV1/FVC < lower limits of normal. Model inputs included age, outpatient or inpatient COPD-related ICD-9 codes, and the number of metered does inhalers (MDI) prescribed over the one year prior to and one year post spirometry. Model performance was assessed using standard criteria.

**Results:**

4564 of 9573 patients (47.7%) had an FEV1/FVC < 0.70. The presence of ≥1 outpatient COPD visit had a sensitivity of 76% and specificity of 67%; the AUC was 0.75 (95% CI 0.74-0.76). Adding the use of albuterol MDI increased the AUC of this model to 0.76 (95% CI 0.75-0.77) while the addition of ipratropium bromide MDI increased the AUC to 0.77 (95% CI 0.76-0.78). The best performing model included: ≥6 albuterol MDI, ≥3 ipratropium MDI, ≥1 outpatient ICD-9 code, ≥1 inpatient ICD-9 code, and age, achieving an AUC of 0.79 (95% CI 0.78-0.80).

**Conclusion:**

Commonly used definitions of COPD in observational studies misclassify the majority of patients as having COPD. Using multiple diagnostic codes in combination with pharmacy data improves the ability to accurately identify patients with COPD.

## Background

Chronic obstructive pulmonary disease (COPD) is a significant cause of morbidity and mortality in the United States and throughout the world [1]. COPD consumes substantial healthcare resources and is among the most expensive medical conditions in the United States [2,3]. Due to the magnitude of the public health and economic burden of COPD, investigators are actively researching all aspects of the genetics, biology, pathophysiology, epidemiology, pharmacotherapy, and healthcare delivery of COPD [4].

The Global Initiative for Chronic Obstructive Lung Disease (GOLD), a partnership between the World Health Organization (WHO) and the National Heart, Lung and Blood Institute, define COPD as the presence of postbronchodilator airflow limitation documented as a fixed ratio FEV_1_/FVC < 0.7 on spirometry [5]. However, there continues to be disagreement among professional societies as to the optimal physiologic criteria to define COPD [6]. While investigators have performed spirometry in population samples quantify the prevalence of COPD throughout the world, such studies are expensive and time consuming to conduct [7-9]. The challenges of obtaining spirometry limit the ability of investigators to identify patients with COPD and investigate differences in COPD care practice across broad geographic regions within the United States.

Many investigators combat these issues by utilizing administrative databases to provide information about the epidemiology of and the care delivered to patients with COPD [10]. The literature is replete with examples of the use of COPD International Classification of Diseases, 9th Revision, Clinical Modification (ICD-9) diagnosis codes to identify COPD cases[10-15] and COPD exacerbations [16-18]. Despite the common use of ICD-9 codes to define COPD in the literature, there is limited data on the validity of such codes [16,19-21]. Prior studies examining the validity of ICD-9 codes utilize medical record review[20,21] or physician diagnosis[16] as the gold standard for COPD. There is limited data characterizing the performance of ICD-9 codes when spirometry is used as the gold standard [22].

We sought to develop a predictive model that would best identify COPD patients using administrative data when spirometry was the gold standard. We focused on determining the performance of outpatient and inpatient ICD-9 codes, but evaluated the ability of additional information, such as age, pharmacy records, and smoking status, to improve the performance of ICD-9 codes in identifying patients with COPD.

## Methods

### Study design

We conducted a secondary analysis of data collected as part of an observational study of medication adherence among patients with COPD.

### Setting and participants

We utilized the Department of Veterans Affairs (VA) inpatient and outpatient databases to screen all patients receiving any inpatient or outpatient care at two VA medical centers in the Pacific Northwest between January, 2003 and December, 2007. We defined the index date when patients entered the sample as the date on which the first pulmonary function test (PFT) including spirometry was performed. We excluded all patients who did not receive postbronchodilator spirometry from our analysis. We also excluded patients with a past or current diagnosis of lung cancer and patients with a BMI < 15 or ≥55 as these patients may have evidence of airflow obstruction for reasons other COPD.

### Data Collection and definitions

We collected demographic data, pharmacy records and the primary ICD-9 code for all outpatient and inpatient visits during the exact calendar date one year pre- and one year post the index date utilizing the VA computerized medical record system.

Patients with any of the following primary ICD-9 codes were considered to have a COPD-related visit: 491.xx - chronic bronchitis, *492.xx - emphysema, 493.2 - chronic obstructive asthma, 496.xx - chronic airway obstruction, not elsewhere classified*. We did not include 4*90 - Bronchitis, not specified as acute or chronic *in our administrative definition of COPD because the definition itself lacks specificity which increases the concern about misclassification [23,24]. Outpatient primary and secondary ICD-9 codes were those recorded during a patient encounter in any outpatient clinic while inpatient primary ICD-9 codes were those recorded during an admission to the hospital. ICD-9 codes generated during visits to the pulmonary function laboratory were not considered in this analysis. Although secondary ICD-9 codes were considered for defining a COPD-related visit these were uncommonly (<7% of visits) coded by providers. Comorbid conditions relevant to patients with COPD were determined using ICD-9 codes for all previous outpatient visits in the one year period prior to the index date. These included a diagnosis of lung cancer (162.x, 163.x), acute coronary syndrome (410.xx, 411.xx), congestive heart failure (398.91,415.xx, 416.xx,425.x, 428.x), diabetes (250.x), hypertension (401.xx-405.xx), atrial fibrillation (427.xx), depression (311, 300.4, 296.2x, 296.3x), and schizophrenia (295.xx).

Smoking was assessed at the time of spirometry; patients were classified as never/former or current based upon self report. We determined the total number of metered dose inhaler (MDI) canisters prescribed over the two year period to each patient for both albuterol and ipratropium bromide (categorized as: albuterol - 0, 1-5, 6+ MDI; ipratropium - 0, 1-2, 3+) using the Veterans Integrated Service Network (VISN) data warehouse. The VISN data warehouse contains the complete pharmacy records for patients who filled prescriptions within the VISN region. These data include the drug name, class, prescription identification number, prescription fill dates (primary and refills), number of allowable refills, date of next allowable refill, amount dispensed, day supply, unit price of the medication and directions for use. Nebulized medications were not included in the calculation of these totals. Tiotropium was not included in our analysis as it was adopted slowly in the VA because of formulary restriction.

### COPD criterion standard

First, we categorized patients using the GOLD criterion: postbronchodilator FEV1/FVC < 0.70 to define COPD. Although experts are actively debating which COPD criterion standard is optimal, GOLD is criticized for identifying false positives among older patients [25,26]. The second definition used a postbronchodilator FEV1/FVC < lower limit of normal (LLN), where the LLN is defined by the referent equations of Hankinson, et al [27]. At both centers, spirometry was performed in accordance with the ATS guidelines for reproducibility [28].

### Statistical analysis

Bivariate comparisons utilized the t-test or Χ^2 ^test as appropriate for the distribution of the variable. We considered a p-value < 0.05 statistically significant.

#### Model development

We pre-specified an approach involving the sequential addition of variables to a multiple logistic regression model for each standard. We considered alternative approaches to model development including classification and regression tress (CART) and neural networks, but neither of these approaches has been consistently shown to outperform logistic regression for most classification problems in medicine [29-31]. We first assessed a model containing a single variable representing the presence/absence of ≥1 outpatient COPD-related ICD-9 code. We then increased the number of codes represented by the indicator variable to ≥3 codes. We then evaluated the performance of ≥1 inpatient COPD-related ICD-9 code. Next, we added pharmacy variables and age to the prior models to characterize changes in the performance of the model after such additions. Because smoking status was assessed by interviewing the patient at the time of spirometry, it is not considered an administrative variable. Nevertheless, this variable may be available to investigators when identifying cohorts of patients with COPD so we added it at the last stage of model development.

We stratified all models by age ≥ 65 years by interacting age (≥65 years) with all variables in each model. This approach allowed separate coefficient estimates for patients ≥ 65 and <65 years of age allowing one to apply the model to Medicare and non-Medicare patients, but provides one number for each estimate of model performance (e.g AUC, Hosmer-Lemeshow, etc).

#### Model evaluation

Model performance was assessed by evaluating the sensitivity and specificity of each model (cut point for the predicted probability of COPD = 0.5), and the discrimination and calibration of each model. Discrimination was determined by calculating the area under the receiver operating characteristic curve (AUC or C-statistic). Calibration was assessed using the Hosmer-Lemeshow goodness-of-fit statistic. We calculated the Brier score for each model as an alternative measure of accuracy that incorporates features of discrimination and calibration as a single measure. The Brier score is calculated as the mean squared error of the model and describes the magnitude by which the predicted probability of COPD generated by the model deviates from the true COPD status of the patient. Because model performance rather than parsimony was our primary concern, we did not employ measures such as the Akaike information criteria during the model building process [32].

For the best performing model containing only administrative variables we determined the sensitivity, specificity, positive and negative predictive values for three cut points (0.25, 0.5 and 0.75) in the model-based predicted probability of COPD. These cut points illustrate the tradeoff in sensitivity and specificity of the model over the range of predicted values. Patients with a model-based probability of COPD greater than the cut point were classified as having COPD. Because the prevalence of COPD in our cohort was high, we also estimated the positive and negative predictive values for the best performing model using prevalence estimates closer to that experienced in the general population (10-20%) [7,9,25].

#### Validation and sensitivity analysis

We utilized the bootstrap (2000 iterations) to internally validate the best performing model. We selected this previously described approach[33] instead of split sample internal validation because it provides an more accurate, unbiased estimate of performance in external cohorts. No external validation was performed.

Finally, we performed two sensitivity analyses to assess the impact of our cohort definition on the results. Because BMI is not captured in most administrative data sources, we re-fit all models after including all patients regardless of BMI. Since the specificity of ICD-9 codes for COPD in younger patients may be low, we also re-fit each model after excluding patients who were < 40 years-old (n = 552).

All analyses were performed using Stata 10.0 (Statacorp, College Station, TX). The institutional review boards for the University of Washington and the participating Veterans Affairs centers approved of the study.

## Results

### Bivariate analysis

We identified 12,205 patients referred for spirometry during the study period. We excluded patients who had a past or current history of lung cancer (n = 330), a BMI < 15 or ≥55 (n = 68), or no assessment of bronchodilator response (n = 2234). After these exclusions, the cohort contained 9573 (78.4%) patients with at least one postbronchodilator assessment (Additional file 1). Among patients assessed, 4564 (47.7%) had fixed airflow obstruction (FEV1/FVC <0.70). Patient demographics, comorbidities, and disease severity are shown in Table 1. Compared to patients with airflow obstruction, patients without airflow obstruction were younger, more often female, and had greater prevalence of diabetes and depression. Patients with obstruction were more likely to be current smokers at the time of PFTs. Patients with fixed airflow obstruction had a lower FEV1 compared to patients without obstruction (1.74 vs. 2.74 L, p < 0.001) respectively. The most common degree of obstruction among patients with obstruction was moderate (48%). Patients with fixed airflow obstruction were also more likely to be prescribed a greater number of MDIs for both albuterol and iprotropium bromide than patients without airflow obstruction.

**Table 1:** Characteristics of cohort by presence of fixed airflow obstruction (GOLD/ATS/ERS standard)

	Cohort (n = 9573)		
		
	No fixed obstruction	Fixed obstruction	p value
Characteristic*	(FEV1/FVC > 0.70)	(FEV1/FVC ≤ 0.70)	
Subjects, n	5009	4564	
Male, n (%)	4567 (91)	4435 (97)	<0.001
Age, years	59 (14)	66 (11)	<0.001
Body Mass Index, n (%)			<0.001
15-18.5	29 (0.6)	135 (3.0)	
18.5-25	714 (14)	1352 (30)	
25-30	1664 (33)	1654 (36)	
30-55	2602 (52)	1423 (31)	
Comorbidities, n (%)			
Hypertension	2667 (53)	2402 (53)	0.55
Diabetes	1174 (23)	792 (17)	<0.001
Congestive heart failure	533 (11)	517 (11)	0.28
Myocardial infarction	101 (2)	113 (2.5)	0.13
Atrial fibrillation	504 (10)	496 (11)	0.20
Depression	1176 (23)	858 (19)	<0.001
Schizophrenia	92 (1.8)	85 (1.9)	0.93
Smoking status, n (%)			<0.001
Never/Former	3616 (72)	2633 (58)	
Current	1393 (28)	1931 (42)	
FEV1, L, mean (SD)	2.74 (0.81)	1.74 (0.74)	<0.001
FEV1, % predicted, mean (SD)	80 (40)	54 (19)	<0.001
Severity of airflow limitation (FEV1, % predicted), n (%)			
GOLD I: Mild (≥80%)	-	458 (10)	
GOLD II: Moderate (50%-79%)	-	2202 (48)	
GOLD III: Severe (30%-49%)	-	1362 (30)	
GOLD IV: Very severe (<30%)	-	541 (12)	
Number of albuterol MDI, n (%)			
0	2925 (58)	1789 (39)	
1-5	1053 (21)	872 (19)	
6+	1031 (21)	1903 (42)	
Number of ipratroprium MDI, n (%)			<0.001
0	3936 (79)	2086 (46)	
1-2	159 (3)	219 (5)	
3+	914 (18)	2259 (50)	

*Numbers represent mean (SD) unless otherwise noted.

FEV1, forced expiratory volume in 1 second; FVC, forced vital capacity; MDI, metered dose inhaler.

### Multivariable analysis

The performance characteristics of the series of models we developed utilizing ICD-9 codes, pharmacy data, age, and smoking status are presented for each reference standard in Table 2. In general, ICD-9 codes by themselves exhibited a modest ability to classify a patient as having airflow obstruction, regardless of the standard (models 1-3), with outpatient codes providing better discriminative ability than inpatient codes (model 1 vs. model 4). Increasing the minimum necessary number of outpatient visits with a primary ICD-9 code for COPD to define airflow obstruction resulted in minimal impact on the AUC beyond that provided by the presence of one or more outpatient diagnostic codes (models 1-3). However, specificity of the model improved when more outpatient ICD-9 codes were required to define obstruction. When added to a model with ≥1 outpatient ICD-9 code, MDI canister counts improved the discriminative ability of the model (models 5, 6). Ipratropium bromide (model 5) appeared to improve the AUC to a slightly greater extent than albuterol (model 6) MDI canisters (AUC 0.77 vs 0.76, respectively). The best performing model utilizing only administrative data (model 8) included the following variables: ≥6 albuterol MDI, ≥3 ipratropium MDI, ≥1 outpatient ICD-9 code, ≥1 inpatient ICD-9 code, and age (model 8, AUC = 0.79, 95% CI 0.78-0.80, Table 2). The overall AUC was qualitatively larger for GOLD standard than for the LLN standard, although changes in the AUC were of similar magnitude when variables were entered into the model. The addition of self reported smoking collected at the time of PFT assessment minimally changed the AUCs and Brier scores for both standards.

**Table 2:** Sensitivity (sens), specificity (spec), discriminative performance (AUC) and calibration (Brier score, Hosmer-Lemeshow [H-L] goodness of fit p-value) for models based on two years of administrative data

			Standard for diagnosis of COPD						
			
			GOLD criterion				LLN criterion		
Model*			(FEV1/FVC < 0.70)				(FEV1/FVC < LLN)		
					
			AUC		H-L		AUC		H-L
#	Input variables	Sens. (%)/Spec. (%)	(95% CI)	Brier	p-value	Sens. (%)/Spec. (%)	(95% CI)	Brier	p-value
1	≥1 outpatient ICD-9 code	76/67	0.75 (0.74-0.76)	0.198		79/62	0.71 (0.70-0.72)	0.198	
2	≥2 outpatient ICD-9 code	70/73	0.75 (0.74-0.76)	0.199		73/69	0.72 (0.71-0.73)	0.196	
3	≥3 outpatient ICD-9 codes	58/83	0.74 (0.73-0.75)	0.201		62/80	0.71 (0.70-0.72)	0.195	
4	≥1 inpatient ICD-9 codes	55/67	0.62 (0.61-0.63)	0.235		6.5/99	0.57 (0.56-0.58)	0.230	
5	≥1 outpatient ICD-9 codes								
	≥1 Ipratropium Bromide MDI	76/67	0.77 (0.76-0.78)	0.192		53/83	0.75 (0.74-0.76)	0.191	
6	≥1 outpatient ICD-9 codes								
	≥1 albuterol MDI	76/67	0.76 (0.75-0.77)	0.196		68/70	0.74 (0.73-0.75)	0.195	
7	≥1 outpatient ICD-9 code								
	≥1 inpatient ICD-9 code								
	≥3 ipratropium bromide MDI								
	≥6 albuterol MDI	68/76	0.78 (0.77-0.79)	0.190		60/79	0.76 (0.75-0.77)	0.188	
8	Model 7 and age	72/74	0.79 (0.78-0.80)	0.187	0.86	60/80	0.77 (0.76-0.78)	0.187	0.50
9	Model 8 and smoking	73/74	0.79 (0.78-0.80)	0.185	0.65	60/81	0.77 (0.76-0.78)	0.185	0.34

* All models are stratified by age ≥65 years. Input variables represent independent variables in the logistic regression equation for that particular model. All variables modeled as dichotomous (present/absent) except for age (continuous).

AUC, area under the receiver operating characteristic curve; H-L, Hosmer-Lemeshow test; LLN, lower limit of normal; MDI, metered dose inhaler

The best performing model incorporating only administrative data (model 8) for both standards was well calibrated (Hosmer-Lemeshow statistic [GOLD p = 0.86; LLN p = 0.50]). The Brier score was lowest for these models as well (GOLD 0.187; LLN 0.187).

Coefficients for the best performing model utilizing administrative data are shown in Table 3. These coefficients are presented for both airflow obstruction standards (GOLD, LLN) and are stratified by age ≥65 years. Table 4 presents the sensitivity, specificity, positive and negative predictive values for various cut points in the model-based predicted probability of COPD generated by model 8 for each diagnostic standard. Utilizing the GOLD standard, setting the cut point in the model-based predicted probability of COPD at 0.25 resulted in a sensitivity (95% CI) of 91% (90-92%), specificity of 41% (39-42%), and positive and negative predictive values of 58% (57-59%) and 83% (81-85%) respectively. Setting the cut point higher in the model-based predicted probability of COPD resulted in greater positive predictive values for both airflow obstruction standards. Estimated PPV and NPV when the prevalence of COPD is closer to population-based estimates (10 or 20%) are presented in the Additional File 2.

**Table 3:** Logistic regression coefficients (beta) for GOLD and LLN diagnostic standard (model 8, table 2)

	GOLD diagnostic standard					
	
	Age < 65 years			Age ≥ 65 years		
	
Variable	Coefficient	95% CI	p value	Coefficient	95% CI	p value
≥1 Outpatient ICD-9 visit	1.266	1.13, 1.40	<0.001	1.394	1.24, 1.55	<0.001
≥1 Inpatient ICD-9 visit	1.262	0.77, 1.75	<0.001	0.575	0.13, 1.02	0.01
≥3 Ipratropium MDI	0.708	0.56, 0.85	<0.001	0.603	0.44, 0.76	<0.001
≥6 Albuterol MDI	0.603	0.47, 0.74	<0.001	0.454	0.29, 0.62	<0.001
Age (per decade)	0.46	0.38, 0.55	<0.001	-0.05	-0.2, 0.1	0.39
Intercept	-4.021	-4.49, -3.55	<0.001	-0.475	-1.33, 0.39	0.28

	**LLN diagnostic standard**					
	
	**Age < 65 years**			**Age ≥ 65 years**		
	
**Variable**	**Coefficient**	**95% CI**	**p value**	**Coefficient**	**95% CI**	**p value**
≥1 Outpatient ICD-9 visit	1.260	1.12, 1.40	<0.001	1.501	1.33, 1.67	<0.001
≥1 Inpatient ICD-9 visit	1.163	0.73, 1.59	<0.001	0.475	0.22, 0.84	0.01
≥3 Ipratropium MDI	0.736	0.59, 0.88	<0.001	0.662	0.59, 0.82	<0.001
≥6 Albuterol MDI	0.606	0.47, 0.74	<0.001	0.509	0.35, 0.66	<0.001
Age (per decade)	0.16	0.07, 0.2	<0.001	-0.28	-0.4, -0.2	<0.001
Intercept	-2.604	-3.03, -2.18	<0.001	0.319	-0.54, 1.18	0.47

MDI, metered dose inhaler; LLN, lower limit of normal

**Table 4:** Sensitivity, specificity, PPV, and NPV for various predicted probabilities of COPD from logistic model 8 by diagnostic standard

GOLD diagnostic standard (model 8)			
	**Model-based predicted probability of COPD (95% CI)**		
	
**Performance characteristic (%)**	**≥0.25**	**≥0.50**	**≥0.75**

Sensitivity	90.9 (90.0-91.7)	71.9 (70.6-73.2)	35.7 (34.3-37.1)
Specificity	40.5 (39.2-41.9)	73.5 (72.3-74.8	92.2 (91.4-92.9)
PPV	58.2 (57.0-59.3)	71.2 (69.9-72.5)	80.7 (78.9-82.4)
NPV	83.0 (81.3-84.5)	74.2 (72.9-75.4)	61.1 (60.0-62.2)

**LLN diagnostic standard (model 8)**			

	**Model-based predicted probability of COPD (95% CI)**		
	
**Performance characteristic (%)**	**≥0.25**	**≥0.50**	**≥0.75**

Sensitivity	83.1 (81.9-84.4)	59.8 (58.2-61.4)	7.5 (6.6-8.4)
Specificity	55.9 (54.6-57.2)	79.6 (78.6-80.6)	98.8 (98.5-99.1)
PPV	54.1 (52.7-55.4)	64.6 (63.0-66.3)	80.1 (75.5-84.2)
NPV	84.2 (83.0-85.3)	76.0 (75.0-77.1)	63.1 (62.1-64.1)

LLN, lower limit of normal; PPV, positive predictive value; NPV, negative predictive value

Bootstrap internal validations of all models resulted in insignificant changes in the AUC and are therefore not reported. Inclusion of all patients regardless of BMI resulted in no substantive changes in all models (data not shown). There were no substantive changes in the models' performance when we limited the cohort to patients over 40 years-old (Additional File 3).

## Discussion

Utilizing over 9500 VA patients with postbronchodilator spirometry we determined that ICD-9 codes have a moderate to good ability to discriminate patients who have fixed airflow obstruction from those who do not with outpatient codes offering better performance than inpatient codes. The addition of a patient's age and pharmacy data including the number of MDIs of albuterol and ipratropium bromide to outpatient and inpatient ICD-9 codes improves the sensitivity and specificity and the overall discriminative performance of a model used to identify patients with airflow obstruction. These variables showed similar performance when utilizing GOLD criteria for airflow obstruction compared to the LLN standard for airflow obstruction.

The use of ICD-9 codes to identify cohorts of patient with COPD using administrative data is common [11-18]. Investigators and payers have utilized these codes to describe the epidemiology of COPD[12,14,15,17,24,34-36], to evaluate the effectiveness and safety of treatments in COPD[11,13,18,37,38], and more recently, as a means to assess the quality of care provided to patients with COPD [16]. In fact, the National Committee for Quality Assurance (NCQA) and the Agency for Health Research and Quality (AHRQ) both advocate for use of quality measures relying on ICD-9 code-based COPD case-identification [39,40]. It is therefore surprising that the validity of both outpatient and inpatient ICD-9 codes for identifying patients with COPD has not been rigorously studied in large populations.

Most prior efforts to establish the validity of ICD-9 codes for COPD utilize chart review or physician consensus as the gold standard. One of the most widely referenced studies, conducted by Rawson and colleagues, utilized the 1987 Saskatchewan health care data files to assess the validity of inpatient COPD ICD-9 codes compared to both the patient's inpatient medical chart and provider service data [20]. Two hundred patient charts were randomly selected from the 4613 hospitalized patients with a primary ICD-9 code for COPD (n = 496). The charted discharge diagnosis from the patient's medical record showed exact agreement for 94.2% of these patients. However, overall concordance between physician documentation of COPD related care and hospital discharge COPD-related ICD-9 codes (490-493, 496) was 68%. An analysis by Ginde and colleagues utilized a similar approach to determine the positive predictive value for principle ICD-9 codes to identify acute exacerbations of COPD in the emergency department [16]. A random sample of 200 patients was taken from all 644 patients with a code for COPD (491.2x, 492.8, 496) at two academic medical centers between 2005 and 2006. Chart review for these patients was used to establish the gold standard for COPD exacerbation which was defined as: 1) the presence of a respiratory infection, 2) change in cough or 3) change in sputum with known physician diagnosed COPD. The overall positive predictive value for the presence of any of the specified codes was 97%. The positive predictive value for a code of 496 alone was 60% (95% CI 32-84%).

Finally, a more recent study using claims in Ontario, Canada examined the combination of ICD-9 outpatient codes and ICD-10 inpatient codes to identify patients with COPD cared for by community providers [19]. The combination of one or more outpatient ICD-9 codes (491.xx, 492.xx, 496.xx) or one or more inpatient ICD-10 codes (J41, J43, J44) had a sensitivity of 85% and specificity of 78.4% among 113 patients with COPD and 329 patients without COPD. An expert panel reviewed each patient's medical record to determine the gold standard for COPD. Spirometry was available in only 180 patients and details about its collection were not reported in the study. The study was further limited by employing ICD-10 codes which have yet to be universally adopted by many countries around the world.

While these studies outlined above suggest that ICD-9 codes can be used to accurately identify physician defined COPD, none universally employed spirometry to define the criterion standard for COPD. Physician diagnosed COPD may not be the optimal gold standard to define COPD. A number of previous studies highlight the difficulty physicians have in correctly indentifying COPD in the absence of spirometry. In North America only 20-30% of patients billed for a COPD-related visit have had spirometry to confirm or refute the diagnosis of COPD [12,41-43]. Up to 20% of physicians confronted with a standardized patient in a COPD exacerbation fail to correctly identify COPD as the cause of respiratory complaints [44]. These data raise concerns about the validity of the COPD gold standard used in prior studies examining the use of ICD-9 codes to identify patients.

The only study utilizing primarily spirometry to define COPD compared discrimination between patients with asthma versus patients with COPD. The accuracy of ICD-9 codes demonstrated excellent performance (AUC 0.98) for the calculated ratio of total COPD ICD-9 codes to total respiratory ICD-9 codes to differentiate patients with asthma from patients with COPD; however, this comparison cannot develop models to predict patients with COPD as the comparator was patients with asthma. Finally, unlike our study, which included over 9500 patients, this study was limited by its inclusion of only 151 patients with COPD [22].

Our study has several strengths. Our gold standard for COPD used the most rigorous definition possible - fixed airflow obstruction on spirometry and captures a large number of patients who had clinical indication for spirometry. This is contrast to many of the previous studies highlighted above.

Our results also have important implications for clinical investigators and health services and health policy analysts. We present the coefficients for a model incorporating administrative variables that can be used to accurately identify patients with COPD. This equation can be used by investigators to calculate the predicted probability of airflow obstruction within novel cohorts. The sensitivity, specificity, positive and negative predictive values for cut points in the model-based predicted probability of airflow obstruction will allow an investigator to maximize sensitivity or specificity depending on the needs of the study practice. For example, one might select a lower cut point (0.25) in the model-based predicted probability of airflow obstruction if utilizing this model to screen a clinical database to identify candidates for a COPD clinical trial. In this situation, maximizing sensitivity would capture the majority of patients with true COPD but at the cost of a large number of false positives. Study staff could access the medical records of these patients to eliminate people without airflow obstruction on spirometry.

We recognize several limitations to our analysis. First, we did not externally validate our model in alternative cohorts of patients. Model performance will likely drop when our model is applied to different patients as a result of geographic and temporal changes, differences in data definitions and case-mix. We assessed the optimism in the estimated AUC for our model utilizing the bootstrap which resulted in no appreciable change in the AUC, but recognize that external validation is a necessary step prior to widespread use [45]. Second, our model was derived on US veterans that were mostly older white men. This may limit the generalizability of our models if applied outside of the VA. In addition, the primary reason for collection of ICD-9 codes in VA patients is not for billing purposes. Differences in coding practice between the VA and other organizations capturing ICD-9 codes primarily for billing purposes may alter the performance of our models if applied outside the VA. Third, some degree of ascertainment bias is likely present, as we were unable to assess clinic visits and hospital admissions to non-VA facilities. Fourth, we collected ICD-9 codes from the one year pre- and one year post the date of spirometry, a time interval that may have reduced the sensitivity and specificity of the codes for COPD. For example, a provider may provide a COPD code on initial evaluation only to learn that spirometry rules out the diagnosis of COPD. Nevertheless, we believe the time interval we used is appropriate because it approximates how ICD-9 codes are screened in observational research and provides a conservative estimate of their performance.

Finally, we limited our cohort to patients referred for spirometry who received a bronchodilator during their test. This was done to ensure that we had a rigorous gold standard by which we defined COPD, but may limit the applicability of our model to only patients who are clinically referred for spirometry. Given the high prevalence of COPD in this population, and the VA more generally [46], the positive predictive value of our model will decrease if applied to a broader population. Several studies suggest that the prevalence of physiologically determined COPD is closer to 10-20%[7,9,25], which is considerably lower than the 48% prevalence observed in our sample. By limiting our analysis to only patients referred to spirometry we provide a conservative estimate of the models performance if applied to a general population. Discriminating patients with COPD from those without COPD among patients who are ill enough to be referred to spirometry is likely a more difficult task than discriminating COPD patients from those without COPD among all patients in a general population. Nevertheless, the estimates of the positive and negative predictive values will change when applying our model to cohorts with different COPD prevalence. Additional testing of our model in broader populations should be done prior to widespread use.

## Conclusion

Administrative data are ubiquitous, are employed in all aspects of healthcare, and are frequently being used to understand the health and healthcare of patients with COPD. Healthcare payers, policy makers, and investigators using administrative data to study COPD rely upon valid assessment of disease status when conducting analyses. Currently used definitions of COPD in observational studies misclassify the majority of patients as having COPD. We determined that ICD-9 codes in combination with pharmacy data can accurately identify patients with COPD. Further validation of our model is required prior to its widespread application.

## Abbreviations

AHRQ: Agency for health research and quality; ATS: American thoracic society; AUC: Area under the receiver operating characteristic curve; BMI: Body mass index; CART: Classification and regression trees; CI: Confidence interval; COPD: Chronic obstructive pulmonary disease; ERS: European respiratory society; FEV1: Forced expiratory volume in one second; FVC: Forced vital capacity; GOLD: Global initiative for chronic obstructive lung disease; ICD-9: International classification of disease, 9^th ^revision; IQR: Interquartile range; LLN: Lower limit of normal; MDI: Metered dose inhaler; NCQA: National committee for quality assurance; PFT: Pulmonary function test: VA: Veterans affairs;

## Competing interests

Dr. Au is a consultant for Nexura, LLC. All other authors have no competing interests to disclose.

## Authors' contributions

CRC designed the study, participated in the analysis interpretation of the data, and drafted the manuscript. MJJ, SMA, and TAL participated in the interpretation of the data and revised the manuscript critically for important intellectual content. EMU and EJ conducted the data analysis, participated in interpretation of the data and revision of the manuscript. DHA received funding for the study, designed the study, participated in the analysis and interpretation of the data, and revised the manuscript. All authors read and approved of the final manuscript.

## Pre-publication history

The pre-publication history for this paper can be accessed here:

http://www.biomedcentral.com/1472-6963/11/37/prepub

## Supplementary Material

Additional file 1 **Study cohort flow diagram**. This figure presents a flow diagram describing the exclusion criteria for the primary analysis. Click here for file

Additional file 2 **Simulated positive predictive values (PPV) and negative predictive values (NPV) for Model 88, by prevalence of COPD**. This table describes how PPV and NPV for Model 8 vary depending on the prevalence of COPD in the population studied. Because the prevalence of COPD in our study population was higher than that in the general population, we simulated what the PPV and NPV would be if the COPD prevalence were 10% to 20%. Click here for file

Additional file 3 **Discriminative performance (AUC) for original models compared to models when cohort limited to patients greater than 40 years-old**. This table illustrates the changes to each model's performance when the cohort excluded all patients <40 years-old. Click here for file
