# Supplementary material for: The validity of using ICD-9 codes and pharmacy records to identify patients with chronic obstructive pulmonary disease
Source: BMC Health Serv Res. 2011 Feb 16;11:37. doi: 10.1186/1472-6963-11-37 (PMC3050695; doi:10.1186/1472-6963-11-37)
Supplement: Additional file 1 — Study cohort flow diagram. This figure presents a flow diagram describing the exclusion criteria for the primary analysis. [file 1472-6963-11-37-S1.DOC]

**Additional File 1**

Study cohort flow diagram.

**Patients referred for spirometry**

(n=12,205)

**Excluded**

Lung cancer (past or current) (n=330)

BMI < 15 (n=24) or ≥ 55 (n=44)

**9573 patients with postbronchodilatory spirometry**

5009 with FEV1/FVC <0.70

4564 with FEV1/FVC ≥ 0.70

**No assessment of bronchodilator response** (n=2234)
