# Supplementary material for: The validity of using ICD-9 codes and pharmacy records to identify patients with chronic obstructive pulmonary disease
Source: BMC Health Serv Res. 2011 Feb 16;11:37. doi: 10.1186/1472-6963-11-37 (PMC3050695; doi:10.1186/1472-6963-11-37)
Supplement: Additional file 2 — Simulated positive predictive values (PPV) and negative predictive values (NPV) for Model 88, by prevalence of COPD. This table describes how PPV and NPV for Model 8 vary depending on the prevalence of COPD in the population studied. Because the prevalence of COPD in our study population was higher than that in the general population, we simulated what the PPV and NPV would be if the COPD prevalence were 10% to 20%. [file 1472-6963-11-37-S2.DOC]

**Additional File 2**

Simulated positive predictive values (PPV) and negative predictive values (NPV) for Model 8 assuming prevalence of COPD is 10 or 20%. The sensitivity and specificity for Model 8 for each model-based probability of COPD cut-point were used to calculate expected PPV and NPV for each standard (GOLD and lower limit of normal) assuming a COPD prevalence of 10% or 20% and a total of 9573 patients in the cohort. Confidence intervals are not presented for estimates of PPV or NPV because data are only simulations.

|  |  | **Model-based (model 8) predicted probability of COPD** | | | | | |
| --- | --- | --- | --- | --- | --- | --- | --- |
|  |  | **≥ 0.25** | | **≥ 0.50** | | **≥ 0.75** | |
|  |  | COPD prevalence | | COPD prevalence | | COPD prevalence | |
| GOLD Standard | | 10% | 20% | 10% | 20% | 10% | 20% |
|  | PPV (%) | 14.5 | 90.9 | 23.2 | 40.4 | 33.7 | 53.3 |
|  | NPV (%) | 97.6 | 40.1 | 96.0 | 91.2 | 92.8 | 85.1 |
| LLN Standard | |  |  |  |  |  |  |
|  | PPV (%) | 17.3 | 32.0 | 24.6 | 42.3 | 41.1 | 61.0 |
|  | NPV (%) | 96.7 | 93.0 | 94.7 | 88.8 | 90.6 | 81.0 |
