# Supplementary material for: The validity of using ICD-9 codes and pharmacy records to identify patients with chronic obstructive pulmonary disease
Source: BMC Health Serv Res. 2011 Feb 16;11:37. doi: 10.1186/1472-6963-11-37 (PMC3050695; doi:10.1186/1472-6963-11-37)
Supplement: Additional file 3 — Discriminative performance (AUC) for original models compared to models when cohort limited to patients greater than 40 years-old. This table illustrates the changes to each model's performance when the cohort excluded all patients <40 years-old. [file 1472-6963-11-37-S3.DOC]

**Additional File 3**

Discriminative performance (AUC) for original models compared to models when cohort limited to patients greater than 40 years-old.

|  |  | **GOLD criterion** | | **LLN criterion** | |  | |
| --- | --- | --- | --- | --- | --- | --- | --- |
|  |  | **(FEV1 / FVC < 0.70)** | | **(FEV1 / FVC < LLN)** | |  | **(FEV1 / FVC < LLN)** |
| **Model** |  | **AUC (95% CI)** | | **AUC (95% CI)** | |  | |
| **#** | **Input variables** | **Original**  **(N=9573)** | **Excluded age < 40 years**  **(N=9021)** | **Original**  **(N=9573)** | **Excluded age < 40 years**  **(N=9021)** |  | |
| 1 | ≥ 1 outpatient ICD-9 code | 0.75 (0.74-0.76) | 0.74 (0.73-0.74) | 0.71 (0.70-0.72) | 0.71 (0.70-0.72) |  | |
| 2 | ≥ 2 outpatient ICD-9 code | 0.75 (0.74-0.76) | 0.74 (0.73-0.75) | 0.72 (0.71-0.73) | 0.71 (0.70-0.72) |  | |
| 3 | ≥ 3 outpatient ICD-9 codes | 0.74 (0.73-0.75) | 0.73 (0.72-0.74) | 0.71 (0.70-0.72) | 0.71 (0.69-0.72) |  | |
| 4 | ≥ 1 inpatient ICD-9 codes | 0.62 (0.61-0.63) | 0.60 (0.59-0.62) | 0.57 (0.56-0.58) | 0.56 (0.55-0.57) |  | |
| 5 | ≥ 1 outpatient ICD-9 codes + ≥ 1 Ipratropium Bromide MDI | 0.77 (0.76-0.78) | 0.76 (0.75-0.77) | 0.75 (0.74-0.76) | 0.74 (0.73-0.75) |  | |
| 6 | ≥ 1 outpatient ICD-9 codes + ≥ 1 albuterol MDI | 0.76 (0.75-0.77) | 0.75 (0.74-0.76) | 0.74 (0.73-0.75) | 0.73 (0.72-0.74) |  | |
| 7 | ≥ 1 outpatient ICD-9 code + ≥ 1 inpatient ICD-9 code + ≥ 3 ipratropium bromide MDI + ≥ 6 albuterol MDI | 0.78 (0.77-0.79) | 0.77 (0.76-0.78) | 0.76 (0.75-0.77) | 0.76 (0.75-0.77) |  | |
| 8 | Model 7 + age | 0.79 (0.78-0.80) | 0.77 (0.76-0.78) | 0.77 (0.76-0.78) | 0.77 (0.75-0.77) |  | |
| 9 | Model 8 + smoking | 0.79 (0.78-0.80) | 0.78 (0.77-0.79) | 0.77 (0.76-0.78) | 0.77 (0.76-0.78) |  | |

* All models are stratified by age ≥ 65 years. Input variables represent independent variables in the logistic regression equation for that particular model. All variables modeled as dichotomous (present/absent) except for age (continuous).

AUC, area under the receiver operating characteristic curve; LLN, lower limit of normal; MDI, metered dose inhaler.
